# Reduced graphene oxide doped tellurium nanotubes for high performance supercapacitor

**DOI:** 10.3389/fchem.2022.1027554

**Published:** 2022-10-18

**Authors:** Pinki Rani, Ashwini P. Alegaonkar, Rathindranath Biswas, Yogesh Jewariya, Krishna Kanta Haldar, Prashant S. Alegaonkar

**Affiliations:** ^1^ Department of Physics, School of Basic Sciences, Central University of Punjab, Bathinda, India; ^2^ Department of Chemistry, Savitribai Phule Pune University, Pune, India; ^3^ Department of Chemistry, School of Basic Sciences, Central University of Punjab, Bathinda, India

**Keywords:** tellurium nanotubes, reduced graphene oxide, doping, electrode material, supercapacitor

## Abstract

Supercapacitors have been achieving great interest in energy storage systems for the past couple of decades. Such devices with superior performance, mainly, depending on the material architecture of the electrodes. We report on the preparation of Tellurium nanotubes (Te-tubes diameter ∼100 nm and length ∼700 nm), with variable doping of conducting network reduced graphene oxide (rGO) to fabricate high-performance electrode characteristics of rGO @ Te. The prepared material was characterized using XRD, FTIR, FESEM, and Raman spectroscopy techniques, including Brunauer-Emmett-Teller, Barrett-Joyner-Halenda measurements. FTIR study revealed that 15% rGO @ Te has a wide C-O vibration band at ∼ 1,100–1,300 cm^−1^, over other compositions. FESEM study shows the Te-tubes dispersion in rGO layers. The EDX study revealed that 15% of the composition has an optimistic concentration of C and O elements. In other compositions, either at lower/higher rGO concentration, an uneven count of C and O is observed. These support efficient charge dynamics to achieve superior ultra-capacitor characteristics, thereby achieving specific capacitance C_sp_ 170 + F/g @ 10 mV/s in a symmetric configuration. The reported values are thirty times higher than pristine Te-tubes (∼5 F/g). This finding suggests that rGO @ Te is a promising candidate for supercapacitor.

## Graphical Abstract

**Figure:**
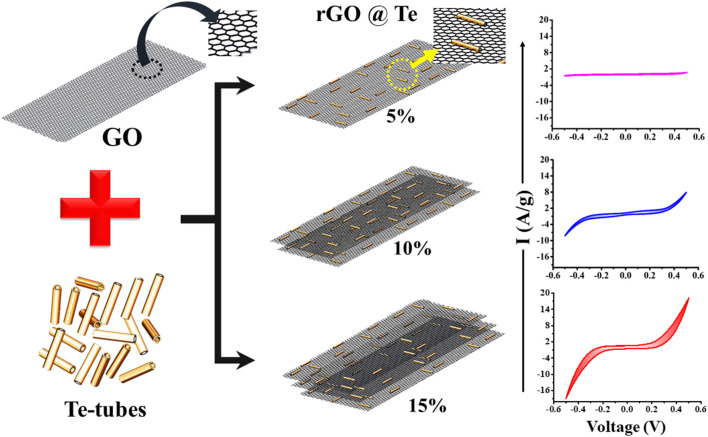
Effect of rGO doping in Tellurium tubes to achieve high electrochemical performance.

## 1 Introduction

Technological improvements are increasing the demand for energy consumption. To some extent, conventional and clean energy resources such as solar energy, wind energy, tidal energy, and geothermal energy are being used to meet the expanding demand for this energy. Even yet energy shortages persist. To satisfy this rising energy demand, researchers are developing novel materials as well as improving the efficiency of existing materials. Supercapacitors are energy sources that are sustainable and ecologically beneficial, and they are used in a wide range of general technical applications. Despite their low energy densities, supercapacitors have high power densities and have around 40,000 charging and discharging cycles (Barik et al., 2022). Supercapacitors are categorised into three varieties based on their charge storage mechanism: electrode double layer capacitors (EDLC), pseudo-capacitors, and hybrids. Electrode materials for supercapacitors are made from carbon, metal oxides/hydroxides, conducting polymers, and transition metal dichalcogenides (Snook et al., 2011; Shi et al., 2014; Cherusseri et al., 2019; Miao et al., 2020). These materials have received extensive research for Supercapacitor applications. As we all know, optimising the electrode material is a critical factor in producing high-quality supercapacitors.

According to the literature, the mono-elemental material such as tellurene, boronene, selenene, and phosphorene are being studied, to enhance the electrochemical characteristics (Tsai et al., 2015; Patil et al., 2016; Li et al., 2018; Ma et al., 2018; Zu et al., 2019; Mariappan et al., 2020; Manoharan et al., 2021). Among them, Tellurium (Te) is an element from chalcogen group with a large atomic radius that has a high theoretical charge storage capacity (420 mA h/g), electrical conductivity (∼2 × 10^2^ S/m), and high material density (6.24 g/cm^3^). All such features make Te a well-suited material candidate for electrochemical performance (He et al., 2017; Wang et al., 2021). Te-based electrodes have been used *via* number of ways such single/bi-/multi-metallic doping in fabricated Te nanostructure to enhance electrochemical performance. Bhol et al. investigated electrochemical performance of Co decorated Te-nanotubes as an electrode material that deliver C_sp_ >140 F/g @ 2 A/g (Bhol et al., 2021). Their asymmetric electrode assembly with activated carbon demonstrated energy density (E_D_) ∼ 51 Wh/kg @ power density (P_D_) ∼ 2294 W/kg. Further improving the energy density Bhol et al. uses bimetallic doping in Te-nanotubes. The (Co-Fe) decorated on Te-tubes electrode material exhibited C_sp_ >170 F/g with an E_D_ ∼ 60 Wh/kg and P_D_ >1000 W/kg (Bhol et al., 2022). Incorporation of transition metal to Te lead to formation various metal tellurides like cobalt, nickel, molybdenum, etc. Manikandan et al. reported the fabrication of cobalt telluride nanorods (CoTe) and Te nanorods. CoTe exhibited C_sp_ ∼ 170 C/g @ 0.5 A/g which was found that higher than Te nanorod 80 F/g (Manikandan et al., 2020). Moreover, they reported nickel telluride to attain C_sp_ > 610 F/g @ 1 A/g (Manikandan et al., 2018). Mao et al. reported nanoflower-shaped cobalt telluride (CoTe_2_) electrode material to achieve C_sp_ > 450 F/g @ 1.5 A/g with excellent cyclic stability (Mao et al., 2020). Composition of Te with other than metals also have been reported, in which Liu et al. studied Te/C nanocomposite for high capacity in lithium–tellurium batteries (Liu et al., 2014). This electrode possessed C_sp_ ∼ 224 mA h/g @ 0.05 A/g. Cao et al. fabricated electrodes Te/Au/MnO_2_ core-shell nanoparticles on carbon fiber that shows C_sp_ > 900 F/g and E_D_ ∼ 36.19 Wh/kg @ P_D_ ∼ 18.61 kW/kg (Cao et al., 2013; Seo et al., 2015).

Therefore, from literature survey, Te based composite material is a potential candidate for supercapacitor application. In the present work, Tellurium tubes use as a host matrix. Generally, Te has self-tendency to grow along a typically crystallographic axis (Chivers & Laitinen, 2015). We prepared Te-tubes by using a wet chemical method. For enhancing the electrochemical performance of Te-tubes, a variable wt% doping of rGO (reduced graphene oxide) is added as a dopant. GO synthesized using Hummer’s method and *in-situ* reduction of GO was carried out along Te-tubes. The electrochemical performance of rGO@ Te showing profitable electrochemical performances. The detail studies of prepared samples and their performance are discussed in detail in present work.

## 2 Experimental

### 2.1 Materials and reagents

The commercially available chemical reagents of analytical grade are used sodium tellurite (Na_2_TeO_3_,99%), sodium molybdate dihydrate (Na_2_MoO_4_.2H_2_O, 98%), sulphuric acid (H_2_SO_4_, 98%), phosphoric acid (H_3_PO_4_), hydrochloric acid (HCl, 37%), hydrazine hydrate (N_2_H_4_.H_2_O), hydrogen peroxide, deionized distilled water (DDW).

### 2.2 Synthesis of tellurium @ reduced graphene oxide: In-situ reduced graphene oxide doping in tellurium

The synthesis of Te-tubes has been carried out using a facile wet chemical method reported previously (Rani et al., 2022). Firstly, 10 mmol of sodium telluride and sodium molybdate were added in hydrazine hydrate and deionized distilled water (in ratio 1:3) solution. The mixture was immersed into a three-neck round bottom flask. This flask was placed into a silicon oil bath, followed by stir for about 5 h @ 120°C. The resultant precipitate was washed with DDW through centrifugation and dried in an oven at 60°C for 12 h.

Further, graphene oxide (GO) was prepared using the Hummer’s method (Emiru & Ayele, 2017). Initially, 0.6 g graphite powder and 4.8 g potassium permanganate were taken in a beaker. In a separate beaker, a mixture of concentrated sulphuric and phosphoric acid with a wt/wt ratio of 9:1 has been prepared. Following this, the mixture of solvent acids was poured slowly into the first beaker. The mixture was stirred for about 12 h @ 40°C in a water bath. Subsequently, in order to stop the reaction, 250 ml of DDW was added to it and 15 ml of hydrogen peroxide was added to the obtain mixture. The obtained solution appeared to be yellow in colour revealing a significant amount of oxidation. The solution was filtered to separate the residue and washed using DDW, ethanol and 5% hydrochloric acid. Finally, samples were dried at 50°C for 24 h with brown colour physical appearance. In order to synthesize the rGO and Te nanocomposite, x wt% of GO, 10 mmol of sodium tellurite and 10 mmol of sodium molybdate were added to the solvent mixture of hydrazine hydrate: DDW = 1:3. For obtaining a homogenous mixture, stirring at 400 rpm has been carried out about half an hour, the solution was poured into a round bottom flask. The flask was placed in an oil bath with constant stir for about 5 h @ 120°C after that black precipitation solution has been formed. Using centrifugation process, the precipitates were washed three times with DDW and dried for about 24 h @ 60°C (vacuum oven). The obtained black sample powder has been collected. Similarly, the initial concentration of x wt% of GO was varied and collected all the sample. In this way, six samples were prepared and designated as TG0, TG1, TG2, TG3 TG4, and TG5, for pristine Te-tubes, and the rest with 5%, 10%, 15%, 20%, and 25% GO, respectively.

### 2.3 Material characterization techniques

The morphology and elemental composition of the sample was analysed using Field emission scanning electron microscopy technique (FESEM, Merlin Compact) with a couple of EDX at an accelerating voltage of 20 kV. The FTIR analysis was studied out using Fourier transform infrared spectroscopy (FTIR, Tensor 27) over a wavenumber range from 600 to 4,000 cm^−1^. The phase and crystal structure of the samples were examined using powder x-ray diffraction technique (XRD, PAN analytical) using Cu, k_α_ = 1.54 Å over 2ϴ range from 0 to 90°. Raman measurement were performed at *λ* ∼ 532 nm. Brunauer–Emmett–Teller (BET) nitrogen adsorption-desorption and Barrett–Joyner–Halenda (BJH) techniques were used to calculate the surface porosity and pore size distribution.

### 2.4 Fabrication of the symmetric supercapacitor assembly

To demonstrate application, supercapacitor electrodes were fabricated using nickel foam as a current collector. To do this, active material, carbon black, polyvinylidene fluoride (PVDF) are taken in a ratio of 70:20:10 and grounded in a mortar -pastel. A slurry has been prepared by addition of two-three drops of N-Methyl-2-pyrrolidone. The resultant homogeneous slurry was pasted onto nickel foam and dried in a vacuum oven @ 60°C for 24 h. The loaded mass over nickel form was ∼1.5 mg. Numbers of electrodes were prepared for the batch of Te, TG1, TG2, TG3, TG4, and TG5. The loading mass kept constant (1.5 mg) for all electrodes. Whatman filter paper was used as a separator, and 6M KOH solution as an electrolyte. A symmetric supercapacitor was prepared by sandwiching two same electrodes with a separator. All electrochemical studies were performed by using NOVA MATLAB 2.1.4 software. Cyclic voltammetry (CV), galvanostatic charge-discharging (GCD) and impedance parameters are measured using a two-electrode system. CV is performed at different scan rates of 10–100 mV/s within a potential window from (−0.5 to 0.5) V. Similarly, GCD was recorded at different current densities.

## 3 Result and discussion

### 3.1 Crystallographic analysis

**FIGURE 1:**
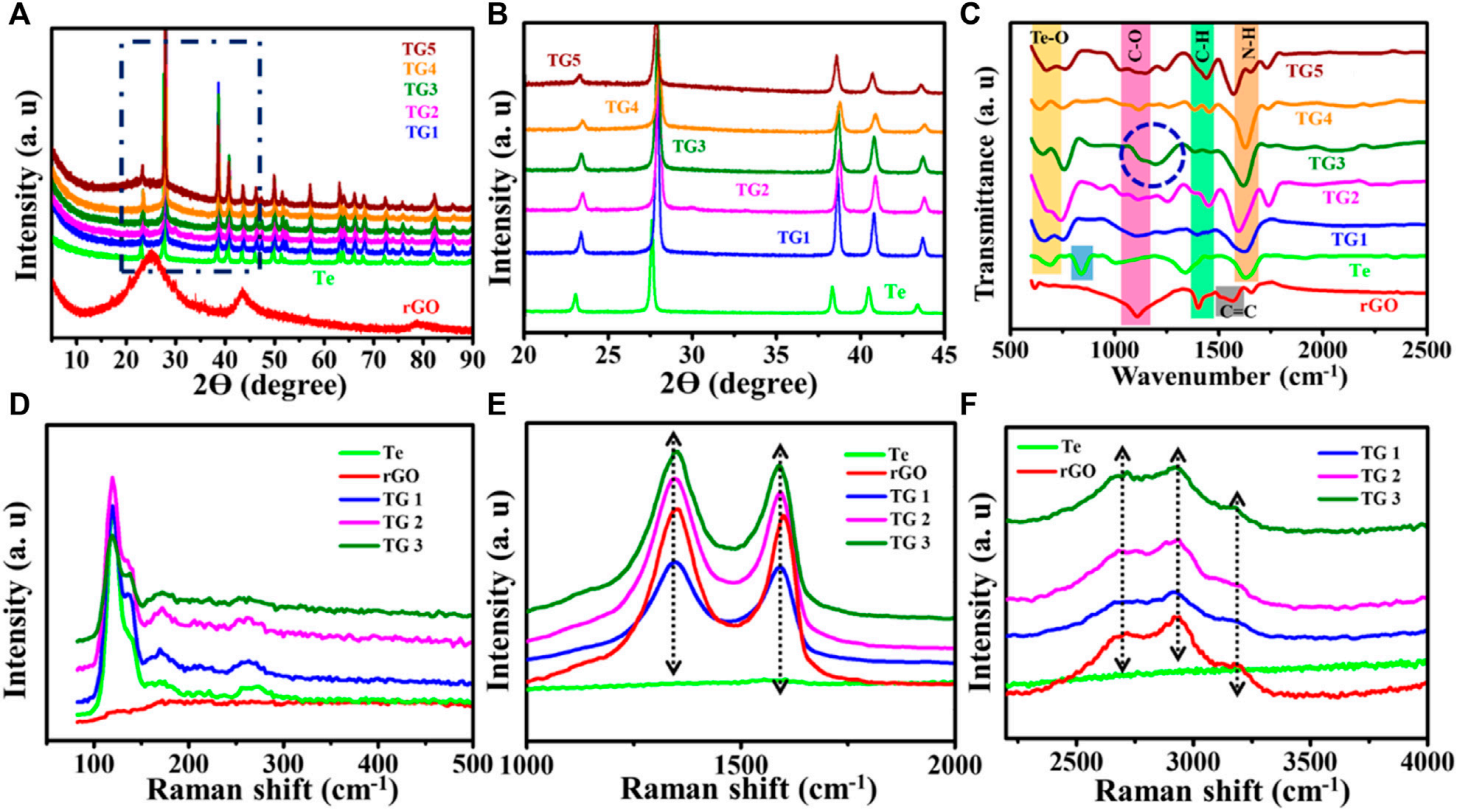
**(A,B)** Recorded XRD data, **(C)** FTIR spectrum, **(D–F)** Raman shift.

Figures 1A,B show recorded XRD patterns for rGO, Te and TG1 to TG5 samples. The XRD of reduced graphene oxide agrees with the previous literature data (Liu et al., 2015; Emiru & Ayele, 2017). Furthermore, the XRD data of synthesised Tellurium is in close agreement with the X’pert high score pdf number 01–086-2269. For Te, the XRD peak at 2ϴ of 23, 27.7, 38.5, 40.63, 43.6, 46.14, 47.31, 49.75, 51.34, 57.163, 63.1, 63.84, 66.063, 67.865, 72.21, 75.81 corresponds to planes (100), (101), (012), (110), (111), (003), (200), (021), (112), (202), (113), (210), (211), (104), (023) and (122) respectively. The peak analysis describes that tellurium has lattice parameters of a, b = 0.4454 nm and c = 0.5924 nm with a hexagonal arrangement. At a low wt% fraction, the presence of rGO in rGO @ Te composite is not noticeable, but, a broad hump appeared below 2ϴ ∼30° is clearly visible for the high content of rGO. In Figure 1B, for 5%–15% rGO@ Te, a small shift towards a higher 2ϴ value signifies contraction of Te lattice planes due to incorporation of rGO within Te-tubes. For Te, the lattice parameter in the c-direction changes somewhat (a, b = 0.4454 nm and c = 0.591 nm), but the diffraction plane remains unchanged. With a higher concentration of rGO in Te, such as 20–25%, the peaks shift again toward a lower 2ϴ. As a result, a subsequent shift in the lattice parameter (a, b = 0.440 nm and c = 0.590 nm) has been observed. Correspondingly modified peak positions, like 23.205, 27.68, 38.439, 40.606, 43.473, 46.035, 49.787 have diffraction planes of (100), (101), (102), (110), (111), (003) and (201), respectively.

### 3.2 Vibrational analysis

Figure 1C displays the FTIR spectra of rGO, Te, and TG 1 to TG5 samples. The band molecular environment of synthesized Te-tube was well explained in our previous report (Rani et al., 2022). For Te, the peak appeared at 667 cm^−1^ is the primary interest that belongs to Te-O bonds (Trivedi et al., 2015). Further, synthesized rGO has four vibration bands appearing at 1,100, 1,403, 1,600, and 1,650 cm^−1^. All rGO peaks agree well with the reported literature (Pai & Nair, 2013). A sharp and prominent peak appeared at ∼ 1,100 cm^−1^, corresponding to C-O stretching vibration modes, together with short small absorbance peaks at ∼1,400, 1,600, and 1,650 cm^−1^, which attributes to C-H, C=C, and bending N-H band group respectively. The small absorbance of C-H and C=C implies that the prepared GO has been partially reduced. For rGO @ Te composite, at 5% a new peak, ∼ 1,150 cm^−1^ has been observed which corresponds to C-OH vibration bands (Gong et al., 2015). For TG1, the emergence of a small peak at ∼ 1,460 cm^−1^, was attributed to C-H bonds (Gong et al., 2015; Wijaya et al., 2020). As the rGO content increases, the peak splits into two peaks within the 1,410–1,470 cm^−1^ wavenumber regime, and finally, a symmetric peak has been seen for TG5. This shows the rearrangement of layers of rGO through Te-tubes. Since the use of hydrazine hydrate as a reducing agent, all of the samples exhibit a peak at 1,630 cm-1, which is indicative of N-H bands. Besides this, a separate peak appeared near the region ∼730 cm^−1^ along with Te-O. This peak corresponds to the Mo-O-Mo bond; which came into the existence due to the presence of sodium molybdate as a precursor material (Fang et al., 2017).

Moreover, for 15% at 1,100–1,300 cm^−1^ region shown by a blue circle, a prominent doublet associated with the C-O stereo functional group is seen. The presence of a significant amount of stereo-regular C-O moiety in the TG3 system may disperse the tubes well within rGO layers. The presence of the C-O band which has donor-loaded characteristics could be favourable for electrochemical characteristics.

### 3.3 Raman studies

**FIGURE 2:**
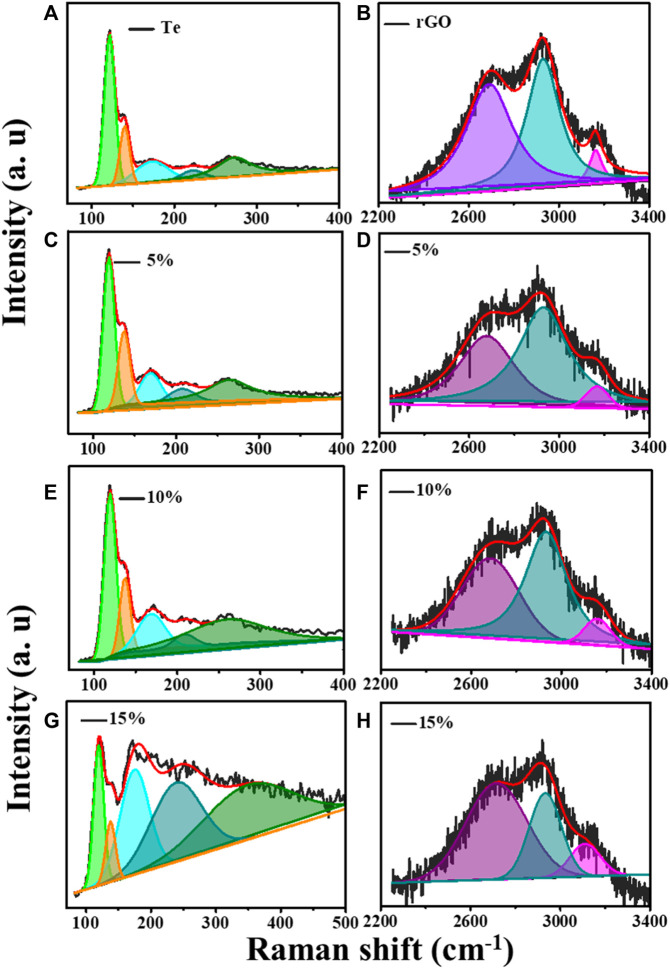
Raman deconvolution in two regions for **(A)** Te tubes, **(B)** rGO, **(C)** and **(D)** for TG1, **(E)** and **(F)** for TG2, **(G)** and **(H)** for TG3.

Figures 1D–F shows Raman spectra over 80–500 cm^−1^, 100–2000 cm^−1^, and 2250–4,000 cm^−1^ for samples Te, rGO, TG1, TG2, and TG3. Four prominent Raman bands has been observed over 100–300 cm^−1^ range, corresponds to Te molecule stretching. With addition of rGO, some new peaks have seen at 1,000–2000 cm^−1^ and 2200–3,300 cm^−1^. These peaks correspond to the stretching vibration of rGO. Raman bands appeared at ∼1,300 cm^−1^ and 1,600 cm^−1^ are associated with carbonaceous D and G shifts. No noticeable shift has been observed in D and G with change in rGO content within Te-tubes. But there are significant variations that has been observed in the 2D (exfoliation), D + G (disorder), and 2D’ (electron-momentum at defect site) Raman bands (Hidayah et al., 2017). A corresponding deconvolution of the Raman spectrum is shown in Figure 2. Table 1 shows the determined parameters such as mode position, FWHM, and intensity for 2D, D + G, and 2D′ Raman shifts. With subsequent increase in rGO content, it was observed that the 2D peak has been shifted to a higher wavenumber region. This indicates the presence of large number of rGO layers. Similar trend for D + G peak signifies increase in disorder.

**TABLE 1:** Raman parameters in higher wavenumber regime.

Raman band	2D			D + G			2D′		
	Position	FWHM	Intensity	Position	FWHM	Intensity	Position	FWHM	Intensity
5%	2676.9	278.622	399.62	2927.98	252.47	562.4	3167.15	124.058	138.56
10%	2682.69	297.82	499.624	2931.72	214.01	669.37	3158.09	137.75	166
15%	2715.48	317.496	586.295	2933.59	166.31	514.55	3110.83	159.89	203.72
rGO	2692.35	235.5	1070.76	2931.72	169.09	1299.3	3163.53	66.98	346.33

The deconvolution of Raman for Te in Figure 2 shows that pristine Te has five stretching shifts: 122.1, 140.5, 172, 221.7, and 273.8 cm^−1^. There are primarily two types of modes has been observed: radial (A1) and axial (E2). Table 2 shows the intensity, position, and FWHM parameters for both modes. The Raman peak at wavenumber 114–123 cm^−1^ corresponds to the A1 mode (Capelo et al., 2021). It is noticed that the incorporation of rGO causes a significant change in the intensity of the A1 modes of vibration.

**TABLE 2:** Raman shift in lower wavenumber.

Raman band	A1			E1		
	Position	FWHM	Intensity	Position	FWHM	Intensity
Te	122.1	15.4	9740.6	140.55	13.66	3759
5%	119.47	16.23	7564.8	137.92	16.87	3918.612
10%	119.47	16.5	5875.74	137.92	15.28	2724.34
15%	119.47	18.34	1431.63	137.92	17.98	615.42

This suggests that, number of sites which are participating in axial vibration process might be reduced significantly. The feature of peak associated with 135–140 cm^−1^ corresponds to E2 (radial) modes (Qin et al., 2020). It shows drastic increase in intensity, and FWHM. This reveals that radial modes of tube dominates planar vibration modes of rGO thereby getting coupled strongly for 15% composition over others. A broad peak at ∼ 172 cm^−1^ and 221 cm^−1^ represent amorphous Te growth ((Vasileiadis & Yannopoulos, 2014; Silva et al., 2017). The Raman peak at ∼ 270 cm^−1^ is attributed to the presence of higher order harmonic modes (E2) of t-Te (Zhang et al., 2009). We have observed higher harmonic shift in E2 modes towards high wavenumber with increase in rGO. It suggests modification in longitudinal stretching of Te-Te bands by rGO planes. This band molecule environment seems to be useful for charge storage characteristics.

### 3.4 Brunauer–Emmett–Teller analysis

**FIGURE 3:**
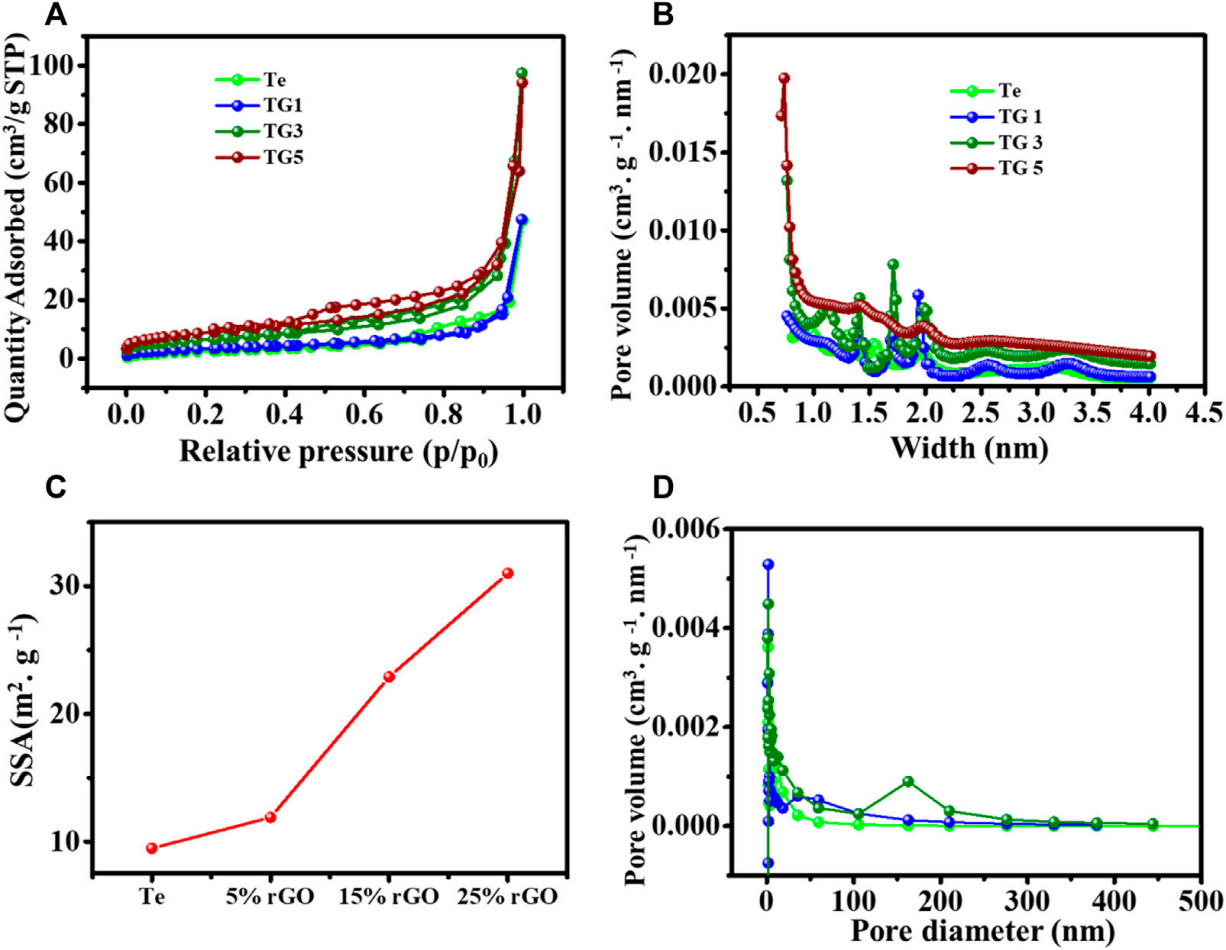
**(A)** Adsorption-desorption isotherm curve, **(C)** determined surface area, **(B,D)** BJH pore size distribution.

To understand the physical properties of the Te and rGO @Te, the nitrogen (N_2_) adsorption-desorption pores size distribution and pore volume were analysed using BET, BJH and HK models, respectively. Figure 3A represented the adsorption isotherm curves for Te and rGO @ Te samples. Both materials possessed the type-III/IV isotherms according to IUPAC meso.

Porosity (Sonal et al., 2020). The hysteresis loop area increases with increasing the content of rGO. Notably, in 25% (TG5), the hysteresis loop area became more significant as it moved towards the low-pressure region @ p/p0 = 0.5; however, TG3 (15%) has an identical hysteresis loop area as that of a high-pressure region. The calculated Langmuir surface area is presented in Figure 3C. It was observed that TG5 and TG3 have surface areas of 31 c and 22.9 m^2^ g^-1^, respectively. For TG1 and TG0, the surface area is lesser as 11.9 m^2^g^−1^ and 9.49 m^2^ g^-1^, respectively. The HK data pore width size distribution is shown in Figure 3B confirms the porous morphology of the synthesized sample. The BJH pore diameter distribution is shown in Figure 3D. The pore size distribution shows that most pores lie in the range of 0–50 nm, indicating micro and mesoporous are present in the sample. Also, it is clear that pore width and diameter are increased with the increased doping of reduced graphene oxide. This could be advantageous for improving electrochemical performance.

### 3.5 Surface morphological investigations

**FIGURE 4:**
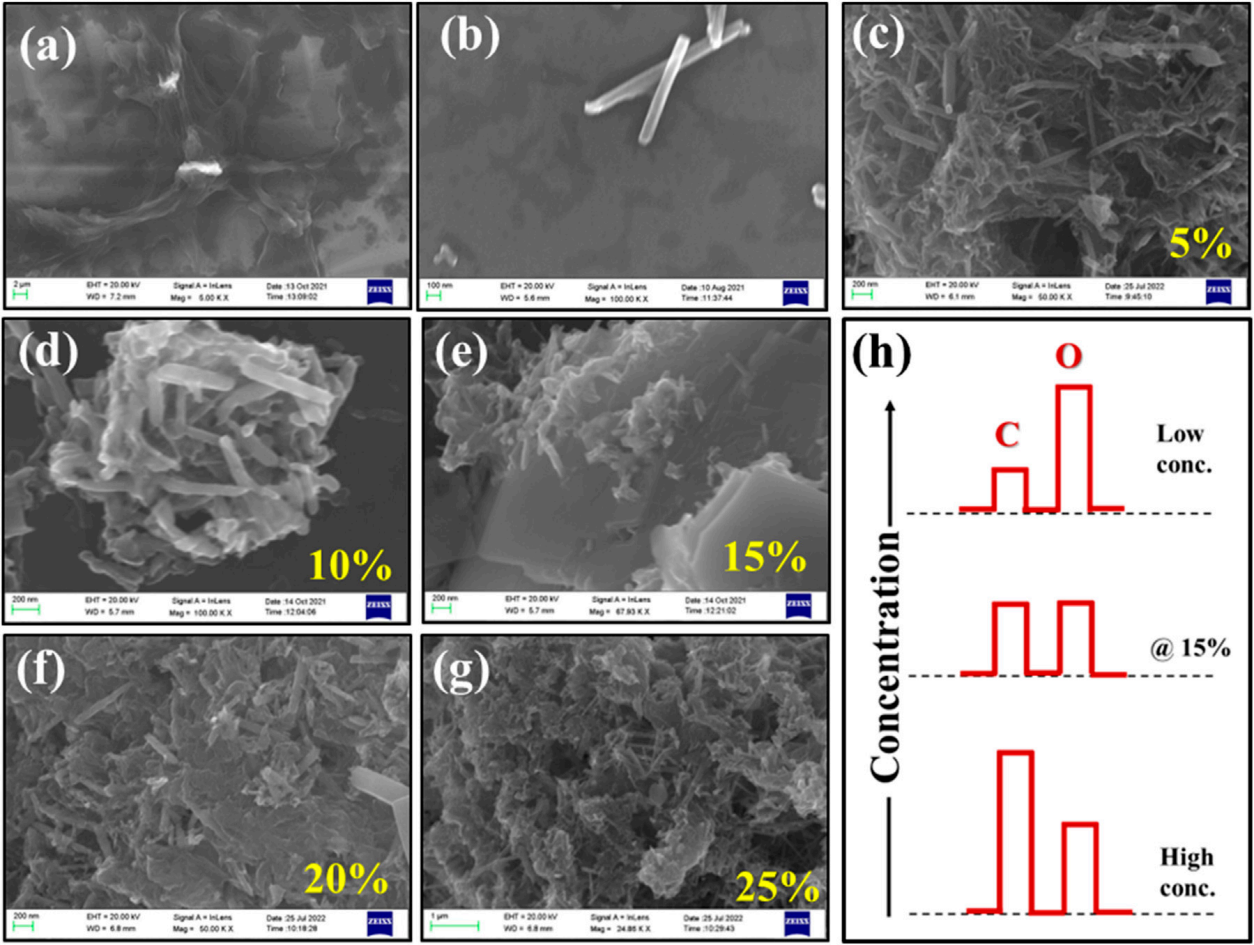
Recorded FESEM images for **(A)** rGO, **(B)** Te, **(C–G)** for TG1, TG2, TG3, TG4 and TG5 respectively; **(H)** Schematic EDS concentration representation for elemental C and O at low to high concentration for rGO @ Te composite.

Figures 4A,B respectively show surface morphology of pristine counterparts of rGO and Te-tubes, whereas Figures 4C–G images shows nature of composite with variable rGO content in Te. Scheme (h) shows concentration variation of element C and O content in rGO @Te composite. From recorded images Figures 4C–G, a systematic change in the surface morphology of the composite has been observed. It shows accommodation of rGO layers, their exfoliation and intercalation with Te-tubes. In image Figure 4C, it is observed that, largely Te-tubes have been spread over rGO scaffolds, however there is no concrete evidence of exfoliation and intercalation. From image Figure 4D as observed that as wt% of rGO increases, Te-tubes might be aggregated forming a larger spherical microstructure. In contrast to Figures 4C,D at 15%, the scaffold of rGO seems to be separated in some places. At higher rGO content i.e., 20 and 25% mostly rupturing of tubes and aggregation of Te-tubes within rGO is seen in images Figures 4F,G. Furthermore, with the help of a bar chart, a corresponding concentration of carbon (C) and oxygen (O) is described in image Figure 4H. It was found, for lower rGO content, the concentration of O is quite high as compared to C content due to variable oxidation characteristics of Te. At low content Te-tubes is unable to exfoliate conjugated rGO layers, whereas at high content the O atoms might be taken away by a number of rGO layers, thereby rupturing Te-tubes. As a result, one can see the morphology displayed in Figures 4F,G are distinctly different compared to the lower concentration image. Somewhat, a balance has been observed at 15% due to commensurate C and O content. This has implication on the degree of exfoliation of conjugate rGO layer; thereby intercalate Te-tube through stereo C-atoms.

### 3.6 Electrochemical parameters

**FIGURE 5:**
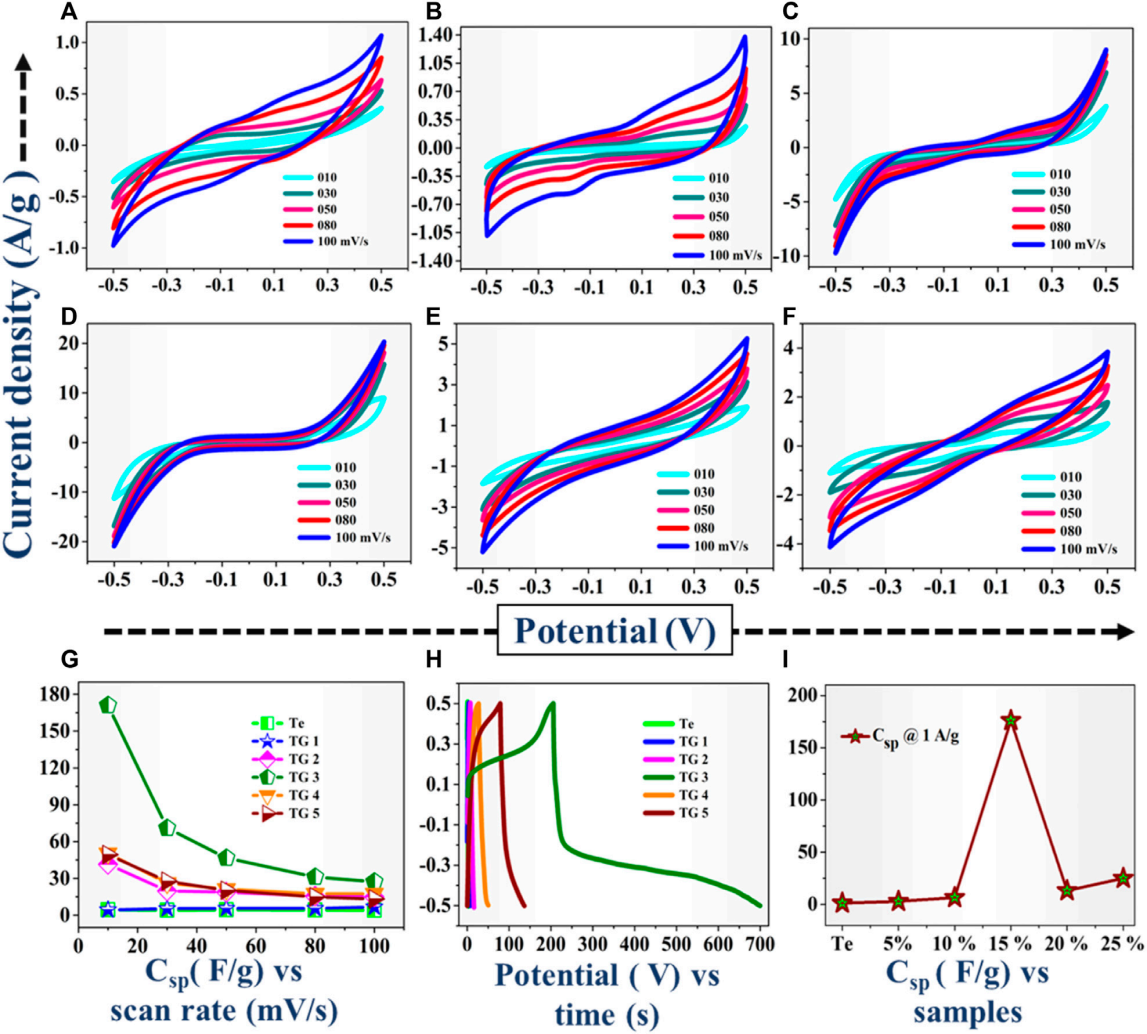
**(A–F)** Recorded CV profiles for Te, TG1 to TG5 (5–25% rGO @ Te) respectively, **(G)** C_sp_ vs. scan rate plot, **(H)** GCD profile @ 1 A/g, **(I)** C_sp_ from GCD.

The electrochemical performance of the Te and 5%–25% rGO@ Te electrode were determined using CV, GCD and impedance spectroscopy techniques. The CV profiles of Te-tubes, TG1 to TG5 samples at different scan rates (10, 30, 50, 80, and 100) mV/s displayed in Figure 5. From CV profiles, it is observed that, the area under the curve is enhanced with the scan rate. The maximum current for pristine Te-tubes is recorded to be 1 A/g. As we increased rGO content from 5% to 15%, the composite exhibited maximum current value of ∼20 A/g. Further increase in rGO content to 20%–25%, found reduction in recorded value of current from 20A/g to 4A/g. From CV plots, the specific capacitance is calculated using the relation (Xiang et al., 2013):

$$C_{sp}=\frac{Area\;under\;the\;curve}{m*v*∆V}$$

Where m is the loading mass of active material, v is the scan rate (mV/s), ∆V is the potential window (volt).

Table 3 and Figure 5G together show the calculated C_sp_ values. For Te-tubes and TG1 to TG5 (rGO @Te composite), with increasing the scan rate, C_sp_ is decreased. At a particular scan rate of 10 mV/s, Te-tube exhibited C_sp_ ∼ 4.47 F/g, and at 15% (TG3) composite achieved highest C_sp_ of 171 F/g. However, subsequent increase in rGO content has decreases the recorded C_sp_ down to 49 F/g. Thus, at 15% composition relatively has higher C_sp_ over others, as displayed in plot Figure 5G. Further, the electrochemical behaviour of the samples was examined by reading GCD characteristics. The recorded GCD @ 1 A/g is shown in plot Figure 5G. From GCD, we observed that for 15%, the discharging is higher than other compositions. The C_sp_ calculated from GCD curve (Pandit, Dubal & Sankapal, 2017; Pandit et al., 2021; Pandit & Sankapal, 2022) using following relation;

$$C_{sp}=\frac{2I\int Vdt}{m\;{\left(V_{f}-V_{i}\right)}^{2}}$$

where I/m is the current density (A/g), ∫Vdt is the area under the discharge curve, V_f_ and V_i_ are the final and initial potential.

**TABLE 3:** Estimated specific capacitance (C_sp_) at variable scan rates for two electrode configurations.

Sample	C_sp_ (F/g) at different scan rates (mV/s)				
	10	30	50	80	100
Te	4.47	4.09	4.46	4.28	4.00
TG 1	4.33	5.31	5.55	5.53	6.68
TG 2	41.5	19.82	18.78	15.6	14.96
TG 3	171.02	70.94	46.6	31.13	27.3
TG 4	50.4	26.14	21.2	17.52	17.42
TG 5	49.38	27.69	20.32	14.89	13.25

The determined C_sp_ @ 1A/g current density is shown in plot Figure 5I. Due to the higher discharging time recorded for 15% composition, it possesses higher value of C_sp_. From both the characteristics, i.e., recorded CV and GCD curves confirms that for 15% rGO @ Te composition has a better electrochemical performance over others. In general, Te has higher magnitude of electrical conductivity than rGO, whereas, the agglomeration of Te-tubes limits their electrochemical performance. As a result, agglomerated Te-tubes does not provide more active sites to participate in electrochemical action. Incorporate rGO into Te-tubes, exfoliated scaffolds rGO thereby intercalation and accommodating Te-tube well in composite. This is responsible to enhance surface and interface area of electrodes. For 15% composition, it seems to be a kind of optimum combination with higher surface area, higher conductivity.

#### 3.6.1 Charge dynamics in reduced graphene oxide @ tellurium

**FIGURE 6:**
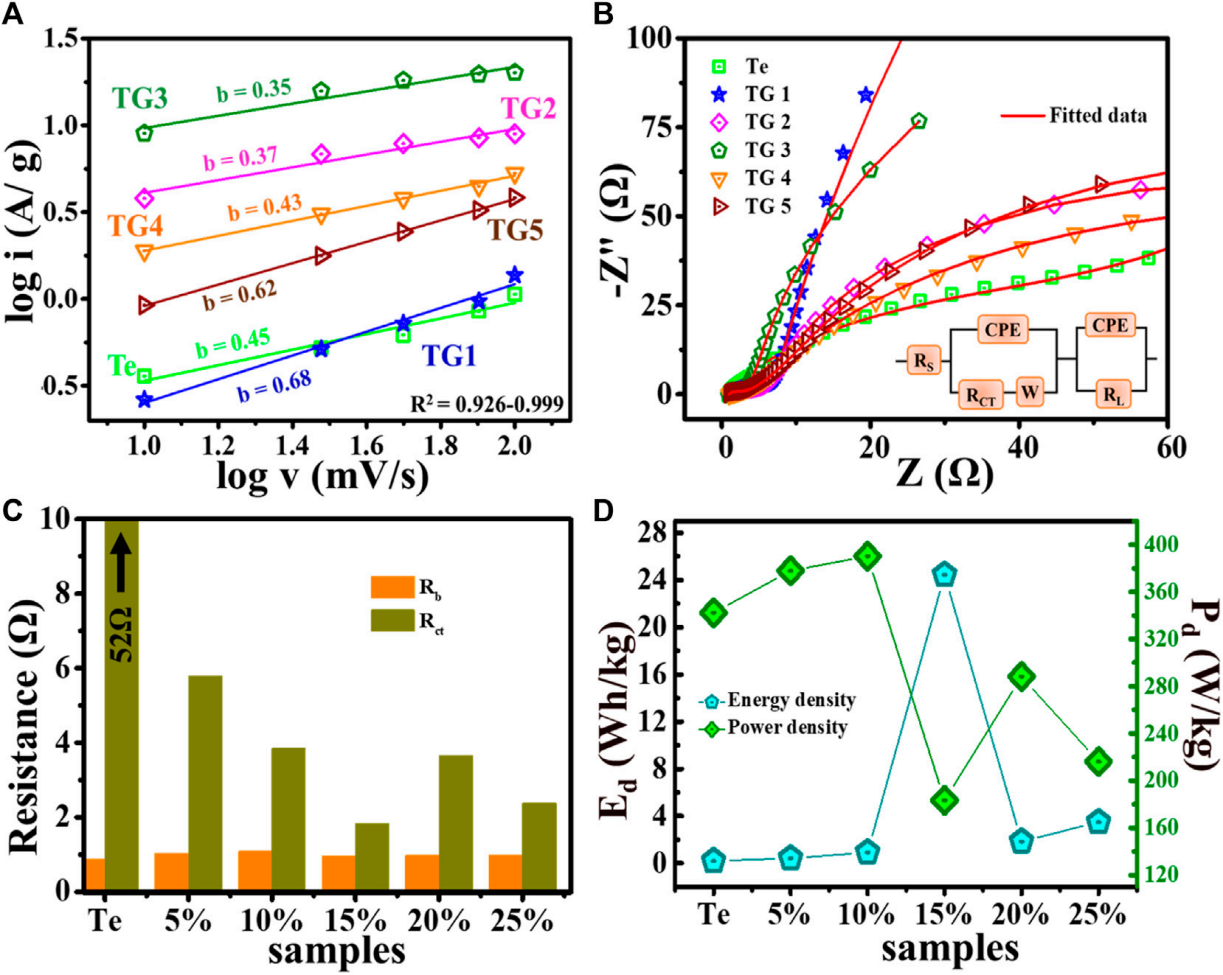
**(A)** log i vs. log v plot, **(B)** Nyquist plot, **(C)** variation in R_b_ and R_ct_, and **(D)** Calculated energy density and power density @ 1 A/g.

We have carried out analysis on mechanism of charge storage over the surface that has been determined with the help of a power law. The contribution of charge storage *via* capacitive or diffusive method have been evaluated by fitting, i = a. v^b^, where i is peak current, v is scan rate, and a and b are the pre-exponent and exponent respectively. The value of b is found from the slope of log i versus log v profiles, as displayed in Figure 6A. The literature (Sankar & Selvan, 2015) revealed that if b is ∼0.5, then the electrode possesses dominating diffusive/intercalative contribution. On the other hand, if the b approaches to 1 then a capacitive behaviour is dictated.

In our study, the value of b for Te-tubes, TG1 to TG5 such as: 0.45, 0.68, 0.37, 0.35, 0.43, 0.62. From CV analysis, at 15% composite showed better electrochemical performance, with b = 0.32, which shows charge storage in 15% composition occurs with dominating intercalated diffusive mechanism. Moreover, electrochemical performance of the fabricated electrodes is analysed using impedance spectroscopy measurements. A Nyquist plot is shown in plot Figure 6B. These obtained experimental profiles are fitted with 3.20 d, demo-version ZSimpWin software. The red line shows the fitted result for impedance, and corresponding simulated circuit is shown in plot Figure 6B inset. The intercept of the Nyquist plot i.e., on the real component of Z (*x*-axis) in the low-frequency region corresponds to total internal resistance (R_s_). The semicircle arc with a straight line has been observed near the high-frequency zone. The charge transfer resistance is represented by the diameter of this semicircle (R_ct_). At high-frequency region, R_s_ and R_ct_ have been calculated for all samples, as shown in Figure 6C. There is no substantial change in R_s_ with the incorporation of rGO in Te-tubes. However, a quick decrease has seen in R_ct_ from 52 + to 2 Ω. From plot Figure 6C, for 15% composition low R_ct_ is recorded indicates superior charge dynamics over other samples.

The constant phase element (CPE) is attributed to the surface homogeneity factor, has been calculated using:

$$Z=\frac{1}{Y_{0}{\left(jw\right)}^{n}};$$

Where Y_0_ is the admittance of an ideal capacitance and the value of n varies from 0 to 1. When n = 1, CPE behave like a pure capacitor, if n = 0 CPE shows resistor like behaviour (Pandit et al., 2019; Goda et al., 2022). The Warburg impedance (W) signifies the impedance that is offered by charges during the diffusion process. The W is seen from the slope around the mid-frequency region (Pandit et al., 2019a). Leakage resistance (R_L_) is demonstrated to be an indicator of the leakage current across the electrode-electrolyte interface (Pandit & Sankapal, 2021). Further, other essential parameters like energy density (E_d_) and power density (P_d_) have been determined with the help of (Pandit et al., 2022a; Pandit et al., 2022):

$$E_{D}=\frac{C_{s}*{\left(V_{f}-V_{i}\right)}^{2}}{2*3.6}$$

$$P_{D}=\frac{E_{D}}{∆t}\times 3600$$

where E_d_ and P_d_ are measure in Wh/kg and W/kg units, respectively (Wang et al., 2018), ∆t is discharge time. The highest E_d_ has been obtained by 15% are ∼24 Wh/kg @ 1 A/g current density and P_d_ was ∼180 W/kg as displayed in Figure 6D. A comparison of electrochemical performance of few other electrode materials has been presented in Table 4. We noted that our performance of rGO @ Te composite in a two-electrode configuration is significantly: higher from Table 4-(1,2,3,5) in C_sp_, comparable to Table 4-(6) in E_D_, comparable to Table 4-(7) in C_sp_ @ three-electrode configuration.

**TABLE 4:** Comparison of electrochemical performance parameter of the supercapacitor based on Te.

S.No.	Electrode materials	Electrode configuration	C_sp_ from CV	C_sp_ from GCD	Energy density (Wh/kg)	Power density (W/kg)	References
1	Te nanowire	Three	24 F/g @ 25 mV/s	—	—	—	Tsai et al. (2015)
2	Te/Au/MnO2	Three	930 F/g @ 2 mV/s	79.15 mF/cm^2^ @ 4 mA/cm^2^	—	—	Cao et al. (2013)
3	Te nanoparticle	Three	93.2 F/g @ 10 mV/s	586 F/g @ 2 mA/cm^2^	116	1,189	Manikandan et al. (2017)
4	P-doped carbon nano-sheet @ Te	Three	—	263 F/g @ 1A/g	36.31	870	Wu et al. (2020)
5	Te and N co-doped carbon	Three	—	197 F/g @ 0.5 A/g	—	—	Kim et al. (2021)
6	CoTe/AC	Three	—	643 F/g @ 1A/g	32.9	800.27	Xiao et al. (2019)
7	Co-Fe decorated on Te	Three	—	179.2 F/g @ 0.9 A/g	62.1	1,138.2	Bhol et al. (2022)
8	rGO doped Te	Two	180 F/g @ 10 mV/s	∼175 F/g @ 1A/g	∼65	500	Our work

We noted that, presence of 15% rGO in Te-tubes has provided a better electrochemical performance atleast by a factor of 35 for C_sp_, ∼25 times for measured E_D_, and improvement in charge transfer tendency by 50 folds. As analyzed, 15% compositions comprised of equal proportion of C and O moieties that acts as an efficient stereoregular functional groups to exfoliate the conjugated rGO layers and coupled the intercalated Te-tubes effectively. The coupling radially transfers the charge to 2D flakes due to dominate radial modes present in Te-tubes.

## 4 Conclusion

We report on the preparation of different wt% of reduced graphene oxide (rGO) @ Tellurium tubes composites to achieve high electrochemical performances. In its basic characterization techniques: XRD study reveals that by incorporating rGO, shrinkage occurs in the Te lattice. FTIR study revealed for 15% at 1,100–1,300 cm^−1^ region a prominent doublet associated with the C-O stereo functional group was observed. The EDX study reveals that only at 15% composition an equivalent amount of C and O atoms has been obtained. From FESEM, it was concluded that the presence of a significant amount of stereo-regular C-O moiety in the TG3 system may disperse tubes well within rGO layers. The C-O bonding is charge donation loaded bonding which could be favourably useful for electrochemical characteristics. Raman studies reveal that rGO disturbs the Te-Te chain longitudinally and disturbs the Te-tubes crystallinity. This amorphous nature of rGO @ Te is favourable for charge storage properties. Also, UV analysis revealed only 15% showing a prominent absorption peak for Te and rGO transitions. The electrochemical study shows the good agreement with basic characterization techniques. As per CV and GCD results 15% of weight percentage of rGO has the higher specific capacitance. Further, increasing the rGO content decreases the current density. It also found that charge storage in rGO @ Te composite mainly occurred *via* an intercalated diffusive process. Therefore, at 15% composition has low charge transfer resistance over others and having excellent charge dynamics.

## Data Availability

The original contributions presented in the study are included in the article/Supplementary Material, further inquiries can be directed to the corresponding authors.
