# Supplementary material for: Reduced graphene oxide doped tellurium nanotubes for high performance supercapacitor
Source: Front Chem. 2022 Oct 18;10:1027554. doi: 10.3389/fchem.2022.1027554 (PMC9623563; doi:10.3389/fchem.2022.1027554)
Supplement: Supplementary file 1 [file DataSheet1.PDF]

Reduced graphene oxide doped tellurium nanotubes for high performance supercapacitor

Pinki Rani[a], Ashwini P. Alegaonkar[b], Rathindranath Biswas[c], Yogesh Jewariya[a], Krishna Kanta Haldar[c], Prashant S. Alegaonkar [a,*]

[a]*Department of Physics, School of Basic Sciences, Central University of Punjab, Bathinda 151401, INDIA*

[b]*Department of Chemistry, Savitribai Phule Pune University, Ganeshkhind Pune 411007 MS, INDIA*

[b]*Department of Chemistry, School of Basic Sciences, Central University of Punjab, Bathinda 151401, INDIA*

Supporting information

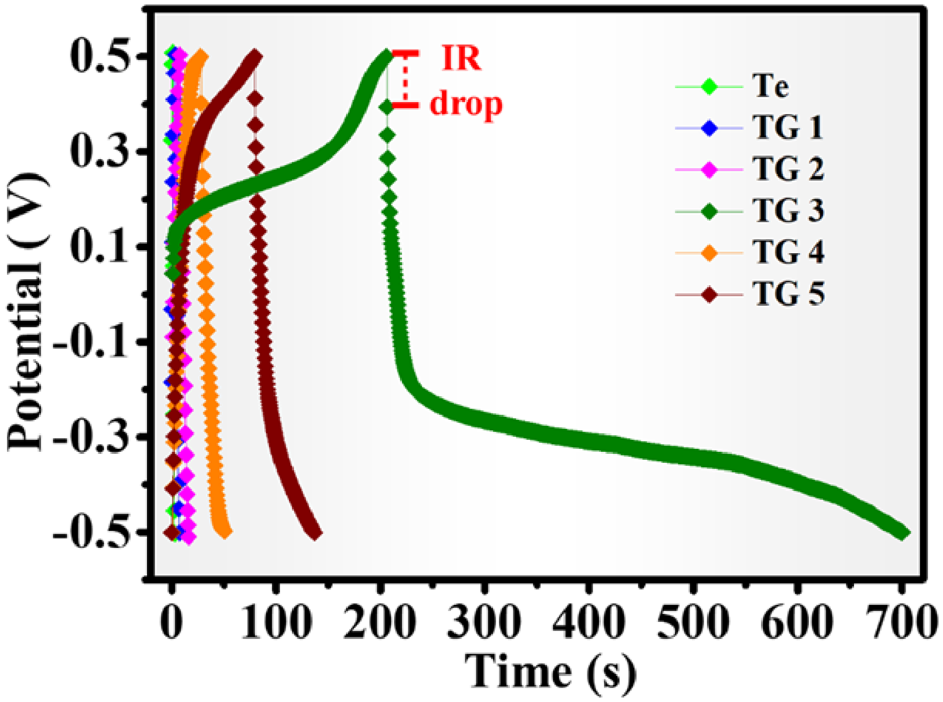

Figure S1: Recorded GCD@ 1A/g.

The estimated values of IR drops are: 0.45 V, 0.22 V, 0.13 V, 0.1 V, 0.097 V, 0.09 V, respectively, for Te, 5%, 10%, 15%, 20% and 25 % rGO @ Te. In our observation, the

* Corresponding author email: prashant.alegaonkar@cup.edu.in

incorporation of rGO has systematically decreased the IR drop. Notably, the drop is almost constant for 15-25% rGO@ Te. Further, it is noted that the calculated IR drop values are in good agreement with previous reports (Huo et al.,2019; Sajjad et al., 2020; Zhao et al., 2015).

The coulombic efficiency calculated using formula:

$$\eta\% = \frac{\Delta t_D}{\Delta t_C} \times 100$$

Δt_D is the discharge time and Δt_D is the charging time. A 15% composition has higher coulombic efficiency (114%) than pristine Te-tubes (72.64%).

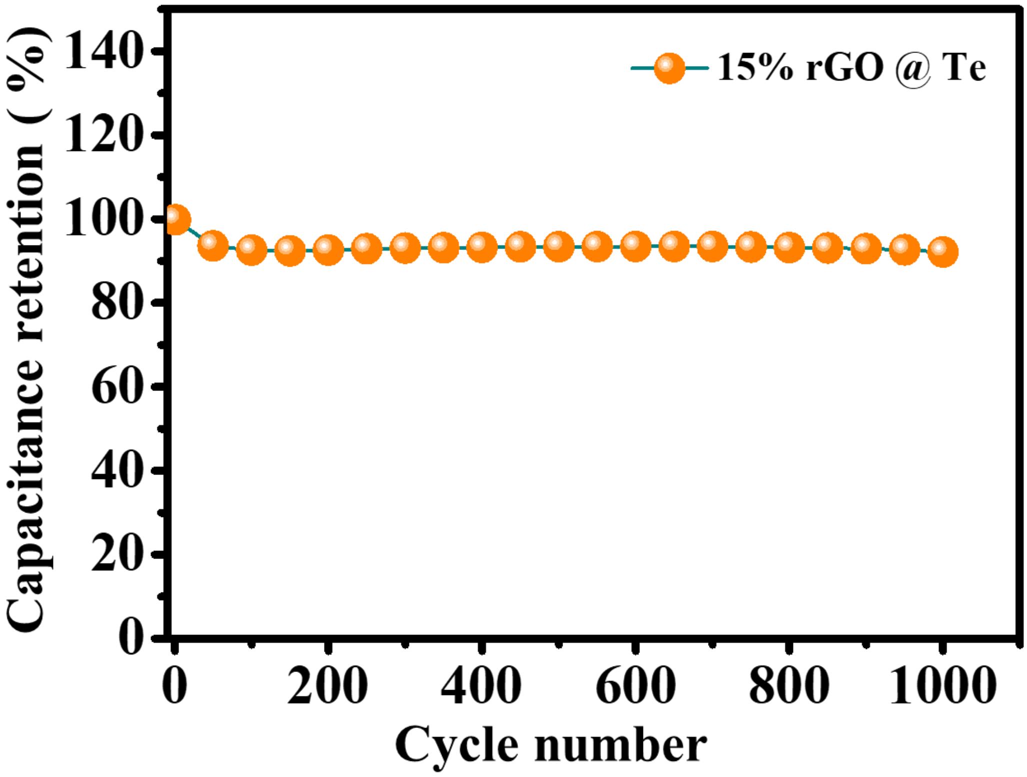

Figure S2: Cycle testing of 15% composition at a 50 mV/s scan rate.

The cyclic stability of supercapacitors was investigated by continuously operating the cyclic voltammetry process under the voltage of -0.5 to 0.5V at a scan rate of 50 mV/s. It was observed that 15% composition shows 92.5% capacitance retention after 1000 cycles.

Reference:

Huo, P., Ni, S., Hou, P., Xun, Z., Liu, Y., & Gu, J. (2019). A crosslinked soybean protein isolate gel polymer electrolyte based on neutral aqueous electrolyte for a high-energy-density supercapacitor. Polymers, 11(5), 863.

Sajjad, M., Xu, C., Guan, L., Zhang, S., Jiao, Y., Zhang, S., . . . Liu, Z. (2020). Influence of Stirring Time on the Electrochemical Properties of NiCo2S4 Hexagonal Plates and NiCo− OH Nanoparticles as High-Performance Pseudocapacitor Electrode Materials. *ChemistrySelect*, *5*(8), 2634-2642.

Zhao, W., He, D., Wang, Y., Hu, Y., Du, X., & Hao, X. (2015). Effects of acid dopants on the capacitance of polyaniline by using graphene hydrogels as substrates. RSC advances, 5(119), 98241-98247.
